# Robotic versus transanal total mesorectal excision in sexual, anorectal, and urinary function: a multicenter, prospective, observational study

**DOI:** 10.1007/s00384-021-04030-5

**Published:** 2021-09-18

**Authors:** Julia-Kristin Grass, Roberto Persiani, Flavio Tirelli, Chien-Chih Chen, Marco Caricato, Alice Pecorino, Isabelle J. Lang, Marius Kemper, Jakob R. Izbicki, Nathaniel Melling, Daniel Perez

**Affiliations:** 1 Department of General, Visceral and Thoracic Surgery, University Medical Centre of Hamburg-Eppendorf, Martinistraße 52, 20246 Hamburg, Germany; 2 Fondazione Policlinico Universitario A. Gemelli IRCCS, Università Cattolica del Sacro Cuore, Rome, Italy; 3 Department of Surgery, Koo Foundation Sun Yat-Sen Cancer Center, Taipei, Taiwan; 4 College of Medicine, National Yang-Ming University, Taipei, Taiwan; 5 Colorectal Surgery Unit, Università Campus Bio-Medico, Rome, Italy

**Keywords:** Functional outcome, Low anterior resection syndrome, Robotic total mesorectal excision, Transanal total mesorectal excision, Urogenital function

## Abstract

**Purpose:**

Improved long-term survival after low anterior resection (LAR) for rectal cancer highlights the importance of functional outcome. Urogenital and anorectal dysfunction is frequently reported after conventional LAR. Advanced minimally invasive techniques such as robotic (RoTME) and transanal total mesorectal excision (TaTME) might improve functional results by precisely dissecting and preserving autonomic nerves. We compared functional outcomes after RoTME or TaTME in a multicenter study.

**Methods:**

One hundred twenty patients (55 RoTME/65 TaTME) were prospectively included in four participating centers. Anorectal (Wexner and low anterior resection syndrome (LARS) Score), urinary (International Consultation on Incontinence—Male/Female Lower Urinary Tract Symptoms Score (ICIQ-MLUTS/ICIQ-FLUTS) and International Prostate Symptom Scale (IPSS)), and sexual (International Index of Erectile Function (IIEF), Female Sexual Function Index (FSFI)) outcomes at 12 months after surgery were compared to preoperative scores. The response rate to the 1-year postoperative functional assessment by questionnaire was 79.5%.

**Results:**

RoTME enabled better anorectal function compared to TaTME (LARS score 4.3 ± 2.2 vs. 9.8 ± 1.5, p = 0.038, respectively). TaTME proved superior at preserving male urinary function, while female urinary function was comparable in both groups, with only mild postoperative impairment (RoTME vs. TaTME, respectively: ICIQ-MLUTS 13.8 ± 4.9 vs. 1.8 ± 5.8, p = 0.038; ICIQ-FLUTS Incontinence Score − 0.3 ± 1.0 vs. − 0.2 ± 0.9, p = 0.844). Both techniques demonstrated comparable male (RoTME − 13.4 ± 2.7 vs. TaTME − 11.7 ± 3.4, p = 0.615) and female (RoTME 5.2 ± 4.6 vs. TaTME 10.5 ± 6.4, p = 0.254) sexual function.

**Conclusion:**

After adjustment for risk factors, RoTME provided better anorectal functional results, whereas TaTME was better at preserving male urinary function. Overall, both techniques demonstrated only mild postoperative functional impairment.

**Supplementary information:**

The online version contains supplementary material available at 10.1007/s00384-021-04030-5.

## Introduction

Over the past three decades, curative treatment of rectal cancer has led to a distinct increase in long-term survival. In addition to earlier diagnosis and more efficient chemoradiation regimes, the introduction of total mesorectal excision (TME) has revolutionized patient outcomes [1, 2]. Sharp dissection of the mesorectal plane enables high rates of negative circumferential and distal resection margins, while precise TME can preserve the autonomic pelvic nerves [3, 4], since these remain outside the dissection plane. As a result, TME has lowered recurrence rates to 5.9% [5], enabling a higher rate of sphincter-preserving procedures [6] and reducing urogenital and fecal dysfunction [4, 7, 8].

However, a substantial subset of rectal cancer patients still suffers from functional impairment after curative rectal resection. Between 40 and 52% of patients state daily bowel dysfunction [9, 10], while one-third of patients develop a long-term urinary dysfunction, shown to be mainly associated with neural damage during surgery [11]. Sexual dysfunction is a multifactorial problem affected by biopsychological and physical factors [4, 12].

Several scores based on patient-reported outcomes measures (PROM) have been established to evaluate and compare functional outcomes. PROMs reflect personal impairment and provide valuable information on the quality of surgery [13]. During the postoperative course, urogenital and anorectal functions decrease to their lowest levels one month after pelvic surgery and recover to stable values after 12 months [9, 14, 15].

Despite several well-known advantages of laparoscopic surgery regarding patient perioperative outcomes and quality of life (QoL), laparoscopic TME (LaTME) has not improved urogenital and anorectal outcomes. Indeed, LaTME has been associated with worse [16, 17] or similar [18, 19] functional results compared to the open approach (OTME). Other advanced minimally invasive techniques, such as robotic (RoTME) or transanal TME (TaTME), can help to master the limitations of conventional laparoscopic rectal resection. Thus, the question arises whether these techniques can also aid in preserving anorectal and urogenital function.

Currently, to the best of our knowledge, no direct comparison of functional outcomes between TaTME and RoTME exists. Thus, our prospective, multicenter, observational study aimed to elucidate which of these two novel techniques would enable better sexual, anorectal, and urinary function.

## Material and methods

### Patient selection

All patients undergoing surgery for primary rectal cancer within 15 cm of the anal verge, either by RoTME or TaTME, between January 2014 and February 2018, were screened for inclusion at the participating institutions. Exclusion criteria were conversion to OTME, other major surgery, disseminated disease, or death in the follow-up period. All eligible patients were invited for participation. The experimental protocol was approved by the responsible review board of the general medical council Hamburg (PV5591). All patients gave their written and signed informed consent to participate in the study.

Clinicopathological and demographic parameters and short-term oncologic outcomes were analyzed. Rectal adenocarcinoma was staged according to the 8th edition of the AJCC staging manual [20]. Adequate local staging was performed by pelvic magnetic resonance imaging (MRI) and/or endorectal ultrasound. In the case of indication for neoadjuvant treatment, patients received long-course chemoradiation with 50.4 Gy and systemic fluorouracil-based chemotherapy. Patients underwent surgery 6–12 weeks after neoadjuvant treatment. A major complication was defined as necessity for further medical or surgical intervention (Clavien Dindo III–IV, invasive treatment) [21]. Anastomotic leakage was defined by any dehiscence at anastomotic site independent of clinical manifestation [22].

### Functional assessment

Validated questionnaires in the respective national language were applied to score the anorectal, urinary, and sexual function at 12 months postoperatively (which is when a stable level of function has been shown to be achieved [9, 14, 15]). Protective ileostomies, if created, were reversed at least 16 weeks before postoperative assessment. These scores were compared to preoperative values collected at time of diagnosis. Questionnaires were sent to the patients or distributed before regular appointments.

Wexner and LARS scores were used to assess fecal function [23, 24]. Patients without a sphincter-preserving procedure were excluded from this analysis. The Wexner score consists of five items and queries continence for solid and liquid stools, as well as gas, the usage of pads, and lifestyle alterations. The maximum score value is 42, representing complete incontinence. The LARS score was developed to evaluate the severity of low anterior resection syndrome (LARS) and includes control of flatus and liquid stool and bowel frequency, as well as clustering of stools and urgency. Patients are summarized in three groups according to their score: no LARS (0–20), minor LARS (21–29), and major LARS (30–42).

Urinary function was evaluated using the International Consultation on Incontinence—Male/Female Lower Urinary Tract Symptoms Score Long Form (ICIQ-MLUTS/FLUTS LF) [25]. The scores are composed of 23 items on male and 18 items on female urinary function, followed by a scale of personal bother measured with a visual analogue scale from 0 to 10. Personal bother scores are not included in the total score, where a higher score indicates more severe urinary dysfunction. Additionally, the International Prostate Symptom Score (IPSS), applied to male and female urinary dysfunction in the literature, was calculated for both genders and evaluated separately [26]. It consists of seven items, yielding a maximum score of 35 points for maximal urinary dysfunction.

Sexual function was analyzed by the International Index of Erectile Function (IIEF) [27] and the Female Sexual Function Index (FSFI) [28]. These self-administered questionnaires are composed of 15 and 19 items, respectively. They query erectile and orgasmic function, sexual desire, intercourse, and overall satisfaction for male patients, and desire, arousal, lubrication, orgasm, satisfaction, and pain for female patients. Higher score values indicate better sexual function.

### Surgery

The participating surgeons had already completed their learning curves of at least 40 cases for either RoTME or TaTME before the start of patient recruitment for this study. One surgeon per site met the inclusion criteria. The surgical procedures have been described previously [29]. In brief, RoTME was performed as a complete robotic procedure using the da Vinci® Surgical System (Intuitive Surgical, Sunnyvale, CA, USA). After dissection of the inferior mesenteric vein (IMV), the splenic flexure and the descending hemicolon were mobilized. Then, the inferior mesenteric artery (IMA) was dissected as a high tie. The pelvic TME was performed by dissecting the mesorectal fascia with identification and preservation of the hypogastric nerves. After transection of the rectum distally of the tumor, the specimen was extracted through a Pfannenstiel incision. TaTME was conducted in a one-team approach in lithotomy position. The abdominal part was conducted as described for the robotic procedure. The TME was conducted up to the level of S3, where the abdominal part was stopped. The transanal approach was started with a purse-string closure of the rectum and insertion of the transanal port. The circular dissection was performed from caudal to cranial connecting the two dissection planes. The specimen was extracted either transanally or via Pfannenstiel.

All anastomoses were performed using a CEEA stapler as side-to-end or end-to-end stapler anastomosis.

### Statistics

All statistical analyses were performed using SPSS version 23 (IBM Corp., Armonk, N.Y., USA). A two-sided p-value of < 0.05 was considered statistically significant. Student’s T-test was used for continuous variables and χ^2^-test for categorical variables. Analyses of pre- and postoperative values were performed with the Wilcoxon test for continuous and categorical variables and McNemar’s test for dichotomous variables.

Traditional covariate adjusted linear models, considered as statistically equivalent to propensity score matching without the bias of observation loss due to exclusion of cases, were used to compare post-treatment change of anorectal and urogenital function between both groups. Anorectal scores have been adjusted for preoperative scores (baseline), body mass index (BMI), gender, tumor height, and radiotherapy. Urogenital scores have been adjusted for preoperative scores, BMI, tumor height, radiotherapy, and restorative surgery.

## Results

### Clinicopathological and short-term outcomes

**Fig. 1:**
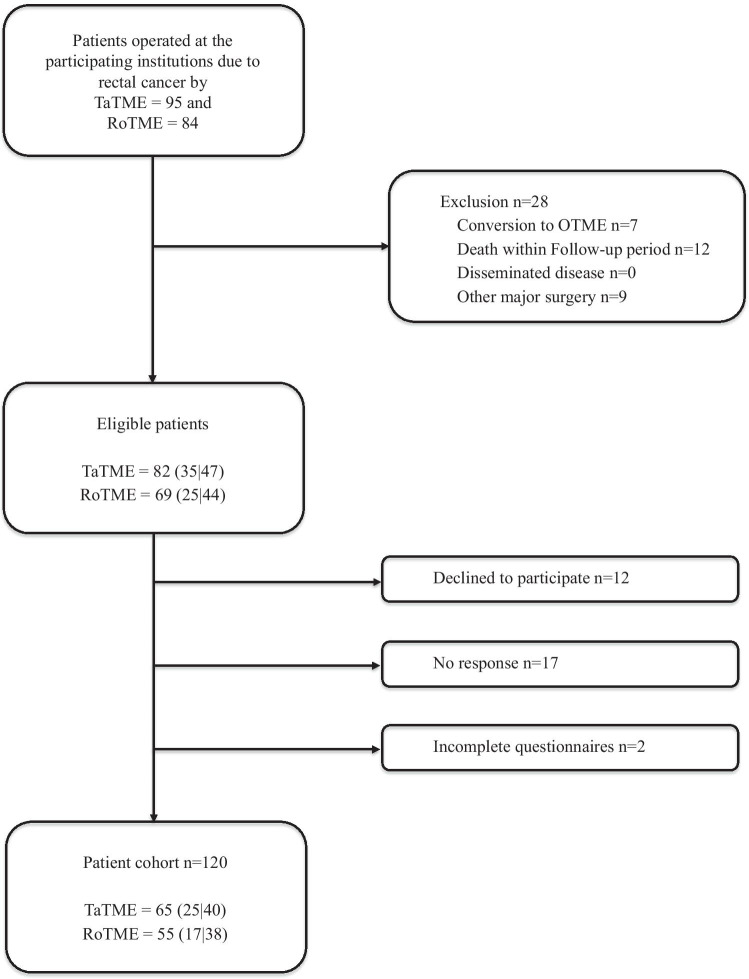
Flowchart of the study populations. Numbers in parentheses indicate female and male patient numbers. OTME, open total mesorectal excision; RoTME, robotic total mesorectal excision; TaTME, transanal total mesorectal excision

One hundred twenty consecutive patients (55 RoTME and 65 TaTME) from four centers were included in the study. The overall response rate was 79.5%: 70.0% for female and 85.7% for male patients (Fig. 1). Thirty-eight (69.1%) RoTME and 40 (61.5%) TaTME patients were male, and the mean age was 59.2 ± 11.9 years in the RoTME cohort and 66.6 ± 10.4 years in the TaTME cohort (Table 1). While there were no significant gender distribution differences, the mean age differed significantly between groups (p < 0.001). There was no significant difference in BMI (27.2 ± 5.3 kg/m^2^ vs. 25.4 ± 4.0 kg/m^2^, p = 0.381) or tumor localization (6.7 ± 5.3 vs. 5.5 ± 2.4, p = 0.116) between the RoTME and TaTME groups, respectively, whereas neoadjuvant chemoradiation was significantly more frequent in TaTME patients (32.7% vs. 63.1%, p = 0.001). The tumor stage was comparable, with pT2–3 stages in most cases (p = 0.182). In contrast, nodal positivity was higher in the RoTME group (pN1 17 (30.9%) vs. 2 (3.1%), p < 0.001). No difference was found in the amount of harvested lymph nodes (p = 0.107).

**Table 1:** Patient characteristics and pathological data

	RoTME (n = 55)	TaTME (n = 65)	p value
Gender			
Male	38 (69.1)	40 (61.5)	0.387
Female	17 (30.9)	25 (38.5)	
Age (years)	59.2 ± 11.9	66.6 ± 10.4	** < 0.001**
Center			
Hamburg	41 (74.5)	0 (0.0)	
Rome Campus Biomedico	0 (0.0)	15 (23.1)	
Taiwan	14 (25.5)	1 (1.5)	
Rome Gemelli	0 (0.0)	49 (75.4)	
BMI (kg/m^2^)	27.2 ± 5.3	25.4 ± 4.0	0.381
Previous abdominal surgery	6 (10.9)	15 (23.1)	0.080
Tumor localization (cm)	6.7 ± 5.3	5.5 ± 2.4	0.116
Neoadjuvant radiochemotherapy	18 (32.7)	41 (63.1)	**0.001**
Tumor stage			
ypT0	6 (10.9)	15 (23.1)	0.182
pT1	13 (23.6)	10 (15.4)	
pT2	18 (32.7)	15 (23.1)	
pT3	18 (32.7)	25 (38.5)	
Nodal stage			
pN0	35 (63.6)	62 (95.4)	** < 0.001**
pN1	17 (30.9)	2 (3.1)	
pN2	2 (3.6)	1 (1.5)	
LN positive (n)	0.8 ± 1.3	0.6 ± 2.2	0.519
LN harvested (n)	16.1 ± 7.9	14.2 ± 5.6	0.107

Numbers indicated as absolute numbers and percentage or mean ± standard deviation; p-values in bold indicate statistically significant differences between surgical techniques

*BMI* body mass index, *LN* lymph nodes, *n* number, *p* p-value, *RoTME* robotic total mesorectal excision, *TaTME* transanal total mesorectal excision

The operative time was shorter for RoTME compared to TaTME (247.0 ± 88.0 min vs. 297.8 ± 85.0 min, p = 0.001) (Table 2). All patients in the TaTME and 87.3% in the RoTME group underwent sphincter-preserving procedures. Protective ileostomies were formed in 70.8% of restorative RoTME and in 100.0% of TaTME procedures. While minor complications occurred more frequently after TaTME (14.5% vs. 32.3%, p = 0.024), major complications were significantly more frequent following RoTME than TaTME (9.1% vs. 0.0%, respectively, p = 0.013), but no significant difference was found for the frequency of anastomotic leakage (8.3% vs. 1.5%, p = 0.117). Resection margin was positive in one RoTME case, with involvement of the circumferential margin (1.8%); however, this did not reach statistical significance (p = 0.275). Aboral and circumferential margins were greater after RoTME compared to TaTME (p < 0.001). Both groups showed comparable quality of mesorectal excision (p = 0.333), as well as similar length of hospital stay (p = 0.166), readmission rates (p = 0.109), and follow-up time (25.9 ± 13.1 vs. 25.7 ± 11.7, p = 0.804).

**Table 2:** Surgical data

	RoTME (n = 55)	TaTME (n = 65)	p value
Operative time (min)	247.0 ± 88.0	297.8 ± 85.0	**0.001**
Estimated blood loss (ml)	38.9 ± 87.5	113.3 ± 252.3	**0.043**
Procedure			
LAR	48 (87.3)	65 (100.0)	**0.003**
APR	7 (12.7)	0 (0.0)	
Minor complication (Clavien-Dindo 1–2)	8 (14.5)	21 (32.3)	**0.024**
Major complication (Clavien-Dindo ≥ 3)	5 (9.1)	0 (0.0)	**0.013**
Anastomotic leakage	4 (8.3)^a^	1 (1.5)	0.117
Resection margin			
R0	54 (98.2)	65 (100.0)	0.275
Aboral R0	55 (100.0)	65 (100.0)	
Aboral margin (mm)	27.2 ± 23.6	10.9 ± 12.3	** < 0.001**
CRM R0	54 (98.2)	65 (100.0)	0.275
CRM margin (mm)	19.0 ± 15.5	4.2 ± 1.3	** < 0.001**
Quality of mesorectal excision			
Grad 1	36 (90.0)	56 (87.5)	0.333
Grade 2	3 (7.5)	8 (12.5)	
Grade 3	1 (2.5)	0 (0.0)	
Length of hospital stay (d)	8.5 ± 4.4	7.3 ± 4.5	0.166
ICU days (d)	0.7 ± 0.8	0.2 ± 0.5	**0.001**
Readmission (d)	7 (12.7)	3 (4.6)	0.109

Numbers indicated as absolute numbers and percentage or mean ± standard deviation; p-values in bold indicate statistical significance between surgical techniques

*APR* abdominoperineal rectal resection, *CRM* circumferential resection margin, *d* days, *ICU* intensive care unit, *LAR* low anterior resection, *min* minute, *ml* milliliter, *n* number, *p* p-value, *RoTME* robotic total mesorectal excision, *TaTME* transanal total mesorectal excision

^a^Percentage of restorative surgery

### Anorectal function

**Fig. 2:**
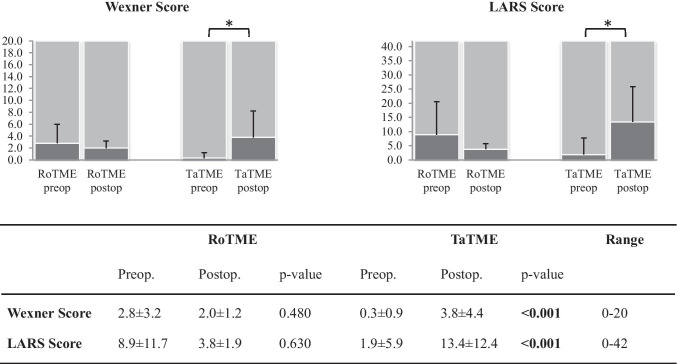
Anorectal function results measured by LARS and Wexner score. Favorable results are at the lower end of the scale. Numbers indicated as mean ± standard deviation; p-values in bold indicate statistical significance between surgical techniques and are indicated on the figure as *. LARS, low anterior resection syndrome; RoTME, robotic total mesorectal excision; TaTME, transanal total mesorectal excision

RoTME patients revealed no significant change in the LARS score (p = 0.630). Comparison of pre- and postoperative scores showed that TaTME led to a highly significant decline in fecal function (Wexner score, p < 0.001; LARS score, p < 0.001), but the actual impairment was mild: the mean Wexner score remained in the lower quarter postoperatively (Fig. 2). After adjustment for potential confounders, Wexner scores revealed no significant difference (Table 3, p = 0.095), whereas the LARS scores indicated better preservation of anorectal function with RoTME (p = 0.038).

**Table 3:** Adjusted model for score differences of anorectal und urogenital function

	**RoTME**		**TaTME**					
	Difference (mean ± SD)	95% CI	Difference (mean ± SD)		95% CI	p-value	Range	Optimum
***Anorectal function***								
Wexner score	1.0 ± 0.7	− 0.485–2.545	2.7 ± 0.5		1.726–3.623	0.095	0–20	⬇
LARS score	4.3 ± 2.2	0.016–8.604	9.8 ± 1.5		6.995–12.786	**0.038**	0–42	⬇
***Male urinary function***								
ICIQ-MLUTS	13.8 ± 4.9	4.008–23.494	1.8 ± 5.8		− 9.749–13.393	**0.038**	1–84	⬇
IPSS	5.1 ± 0.9	3.287–6.928	0.3 ± 1.0		− 1.677–2.376	** < 0.001**	0–35	⬇
**Female urinary function**								
ICIQ-FLUTS—Filling Score	1.4 ± 1.7	− 2.190–4.933	− 1.1 ± 1.6		− 4.490–2.293	0.050	0–15	⬇
ICIQ-FLUTS—Voiding Score	− 1.0 ± 0.7	− 1.631–1.440	0.7 ± 0.8		− 0.888–2.294	0.185	0–12	⬇
ICIQ-FLUTS—Incontinence Score	− 0.3 ± 1.0	− 2.347–2.090	− 0.2 ± 0.9		− 2.155–1.664	0.844	0–20	⬇
IPSS	0.1 ± 2.2	− 4.513– − 4.745	0.0 ± 1.9		− 3.972–4.041	0.961	0–35	⬇
***Male sexual function***								
IIEF—erectile function	− 1.5 ± 1.2	− 3.818–0.881	− 1.7 ± 1.4		− 4.462–1.162	0.902	0–30	⬆
IIEF—orgasmic function	− 3.8 ± 0.6	− 4.968– − 2.609	− 2.7 ± − 0.8		− 4.162– − 1.115	0.120	0–10	⬆
IIEF—sexual desire	− 1.9 ± 0.5	− 2.859– − 1.048	− 1.5 ± 0.6		− 2.662– − 0.405	0.441	0–10	⬆
IIEF—satisfaction sexual intercourse	− 3.7 ± 0.7	− 5.139– − 2.313	− 2.7 ± 0.9		− 4.481–0.900	0.236	0–15	⬆
IIEF—overall satisfaction	− 2.4 ± 0.5	− 3.349– − 1.419	− 1.8 ± 0.6		− 3.033– − 0.719	0.368	0–10	⬆
IIEF—final score	− 13.4 ± 2.7	− 18.824– − 7.882	− 11.7 ± 3.4		− 13.911–0.052	0.615	0–75	⬆
***Female sexual function***								
FSFI—desire	0.3 ± 0.6	− 0.872–1.408	0.7 ± 0.7		− 0.656–2.089	0.317	1.2–6	⬆
FSFI—arousal	− 0.0 ± 0.8	− 1.640–1.632	0.5 ± 1.0		− 1.558–2.486	0.532	0–6	⬆
FSFI—lubrication	1.4 ± 0.9	− 0.447–3.174	2.6 ± 1.2		0.226–4.995	0.100	0–6	⬆
FSFI—orgasm	1.2 ± 1.0	− 0.840–3.247	1.7 ± 1.3		− 0.874–4.356	0.516	0–6	⬆
FSFI—satisfaction	0.5 ± 0.8	− 1.116–2.095	1.4 ± 1.0		− 0.661–3.535	0.218	0.8–6	⬆
FSFI—pain	1.9 ± 0.9	− 0.017–3.842	3.8 ± 1.2		1.280–6.362	**0.043**	0–6	⬆
Final FSFI	5.2 ± 4.6	− 4.335–14.813	10.5 ± 6.4		− 2.783–23.868	0.254	2.0–36.0	⬆

Adjusted linear models for preoperative functional score, BMI, tumor height, radiotherapy, gender for anorectal scores, and for baseline, BMI, tumor height, radiotherapy, restorative surgery for urogenital scores. Values shown as mean ± standard deviation for pre- and postoperative score differences and confidence interval; p-values in bold indicate statistical significance between surgical techniques, and arrows indicate optimum function at upper or lower score range. For anorectal and urinary function, positive difference values indicate a postoperative deterioration, while for sexual function, negative difference values represent a postoperative deterioration

*BMI* body mass index, *CI* confidence interval, *FSFI* Female Sexual Function Index, *ICIQ-MLUTS/ICIQ-FLUTS* International Consultation on Incontinence—Male/Female Lower Urinary Tract Symptoms Score, *IIEF* International Index of Erectile Function, *LAR* low anterior resection, *RoTME* robotic total mesorectal excision, *SD* standard deviation, *TaTME* transanal total mesorectal excision

### Urinary function

**Fig. 3:**
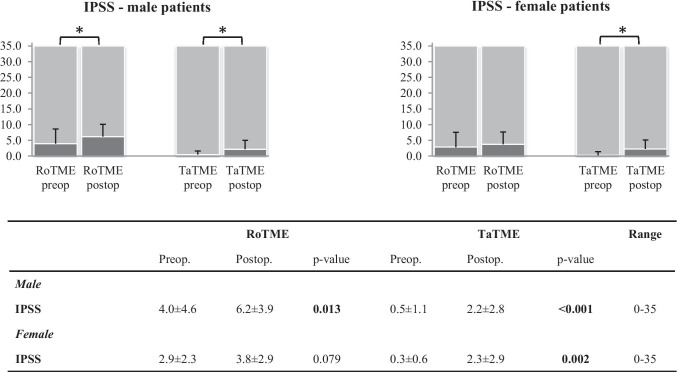
Urinary function measured by IPSS. Favorable results are at the lower end of the scale. Numbers indicated as mean ± standard deviation; p-values in bold indicate statistical significance between surgical techniques and are indicated on the figure as *. IPSS, International Prostate Symptom Score; RoTME, robotic total mesorectal excision; SD, standard deviation; TaTME, transanal total mesorectal excision

Both RoTME and TaTME, respectively, led to mild but significant postoperative impairment of male urinary function (IPSS p = 0.013 vs. p < 0.001 (Fig. 3); ICIQ-MLUTS p = 0.032 vs. p < 0.001 (Online Resource Fig. 1)), with postoperative mean values still lying in the top quarter of the respective range. The adjusted analysis of pre- and postoperative score differences revealed significantly lower urinary tract dysfunction in the TaTME group for ICIQ-MLUTS and IPSS score (IPSS RoTME 5.1 ± 0.9 vs. TaTME 0.3 ± 1.0, p < 0.001; ICIQ-MLUTS RoTME 13.8 ± 4.9 vs. TaTME 1.8 ± 5.8, p = 0.038 (Table 3)).

Female urinary function was also impaired after surgery. According to IPSS, female RoTME patients had preserved function, while TaTME patients revealed significant postoperative deterioration (p = 0.002, Fig. 3). Nevertheless, postoperative impairment was again very mild, since the mean values remained in the lower 10–20% of the score range. Moreover, RoTME patients had a preserved ICIQ-FLUTS voiding score (p = 0.480), but filling (p = 0.017) and incontinence scores (p = 0.027) worsened postoperatively. For TaTME, all ICIQ-FLUTS dimensions were significantly affected (p = 0.007, 0.027, and 0.041, respectively; Online Resource Fig. 1). The adjusted analysis revealed no significant differences between techniques for female bladder function (IPSS p = 0.961; ICIQ-FLUTS p = 0.050, 0.185, and 0.844, respectively (Table 3)). Only the ICIQ-FLUTS filling score came close to statistical significance, with a trend in favor of TaTME (p = 0.050).

### Sexual function

**Fig. 4:**
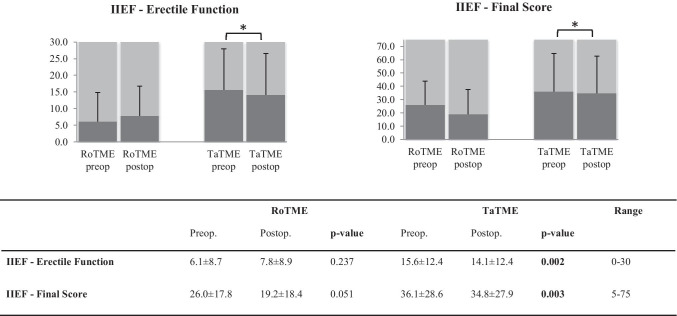
Male sexual function measured by IIEF. Favorable results are at the upper end of the scale. Numbers indicated as mean ± standard deviation; p-values in bold indicate statistical significance between surgical techniques and are indicated on the figure as *. IIEF, International Index of Erectile Function; RoTME, robotic total mesorectal excision; TaTME, transanal total mesorectal excision

Measured by IIEF, male sexual function was slightly impaired after TaTME: erectile function (p = 0.002), IIEF final score (p = 0.003, Fig. 4), and satisfaction with sexual intercourse (p = 0.034, Online Resource Fig. 2) demonstrated significant postoperative impairment. RoTME patients had comparable pre- and postoperative results regarding male sexual function. Overall, after adjustment for confounders, both techniques equally revealed a low impact on male sexual function (Table 3).

**Fig. 5:**
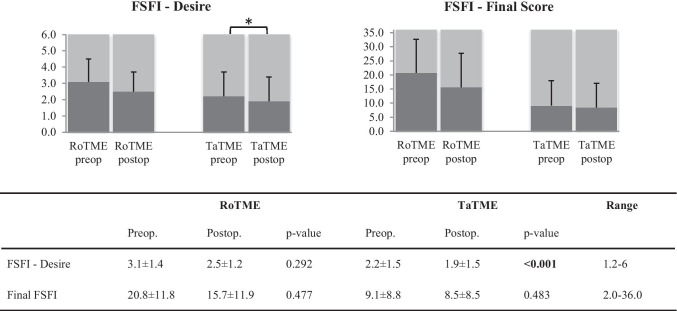
Female sexual function measured by FSFI. Favorable results are at the upper end of the scale. Numbers indicated as mean ± standard deviation; p-values in bold indicate statistical significance between surgical techniques and are indicated on the figure as *. FSFI, Female Sexual Function Index; RoTME, robotic total mesorectal excision; TaTME, transanal total mesorectal excision

Female sexual desire was significantly impaired after TaTME (p < 0.001) (Fig. 5), but the adjusted comparison of individual pre- and postoperative values showed comparable results for RoTME and TaTME in this FSFI dimension. All other FSFI subscores proved equal for both techniques (Online Resource Fig. 3), apart from the pain score, which was significantly lower in the TaTME group (p = 0.043, Table 3).

## Discussion

RoTME can preserve anorectal function better than TaTME. In contrast, TaTME is superior regarding male urinary function and may have advantages for female urinary outcomes. For sexual function, comparable results were demonstrated for both techniques; this may result from the multifactorial etiology of sexual dysfunction, on which surgery has the smallest impact [30, 31].

Besides physical affection during surgery, several factors might influence functional outcome after TME. Radiotherapy induces neural damage and thereby impairs bowel function via deterioration of neorectal sensitivity [32]. Moreover, it leads to erectile dysfunction and causes hypogonadism in male and female patients [30, 33]. Tumor height also impacts functional outcomes. The further the TME plain has to be dissected towards the pelvic floor, the higher the risk for neural damage. Moreover, gender, and preoperative functional status influence the postoperative anorectal and urogenital outcomes [10, 34]. Hence, postoperative results must be studied in light of these factors. Therefore, we conducted an adjusted linear model, considering all parameters as adjustment factors.

Anorectal dysfunction remains frequent after LAR, with a reported rate of 46.1% [35] for major LARS using the open approach and increasing after chemoradiation [10]. Likewise, LaTME is associated with major LARS in 29.6–52.0% of cases [36, 37]. Deterioration of bowel function is caused by damage to the internal anal sphincter, which induces impairment of passive anorectal continence, and the hypogastric plexus.

Our analysis reveals no significant increase compared to the preoperative score for RoTME. In contrast, TaTME patients reported significant postoperative impairment of anorectal function. Nevertheless, worse fecal function was previously shown for TaTME, with major LARS ranging from 33.3 to 59.3% [6, 36–38], and was inferior to LaTME in direct comparisons in several series [6, 38, 39]. Contributing factors might be lower anastomoses due to technical implications and damage to the internal sphincter by prolonged dilation. Moreover, an elevated risk of entering the extramesorectal plane during the transanal phase may be accountable for a higher proportion of autonomic nerve injuries following TaTME [37]. In our series, after adjustment for confounders (radiation, preoperative anorectal function score, gender, BMI, and tumor height), RoTME resulted in significantly better anorectal function compared to TaTME.

Several non-randomized studies have revealed better urogenital function after RoTME compared to LaTME [14, 40], whereas the randomized ROLARR trial showed no difference in bladder or sexual function between LaTME and RoTME [41]. These findings are limited by a short surveillance period of only 6 months and the fact that participating surgeons, while experienced in LaTME, may still have been in their learning curve for RoTME. A recent meta-analysis implied superior urogenital results for RoTME over LaTME [34]. Furthermore, TaTME has been shown to provide optimized urinary and male sexual function with greater experience compared to earlier published results [6, 39].

Urinary dysfunction is mainly caused by surgical nerve damage [11]. Sympathetic nerve injury induces bladder instability, whereas parasympathetic neural damage triggers detrusor instability [31], causing urge incontinence and voiding dysfunction. Interestingly, evidence on the negative impact of radiation is missing [30]. The IPSS, originally designed for benign prostate hyperplasia [26], is widely accepted for assessing urinary dysfunction in male and female rectal cancer patients, but its application following rectal surgery remains questionable. In contrast, the ICIQ-MLUTS and ICIQ-FLUTS have been developed as universally applicable questionnaires [25]. Male patients experienced a mild, but significant decrease in urinary function in both scores after RoTME and TaTME, in line with the literature [14, 15, 37, 38]. Similarly, a significant deterioration of all urinary functional scores was found in female patients. Only female RoTME patients had stable ICIQ-FLUTS voiding scores (p = 0.480). After adjustment for preoperative score values, BMI, radiation, tumor height, and restorative surgery, TaTME demonstrated better urinary results than RoTME for male patients, while female urinary function did not show any difference. Improved exposition of hypogastric nerves might be an advantage of TaTME, leading to superior urinary outcome.

In contrast, sexual dysfunction is a multifactorial issue, which is also influenced by bio-psychological factors such as poor self-image, depression, fatigue, loss of independence, and changes in personal relationships. Surgical aspects in this context include neural damage, cosmetic appearance and stoma fashioning [30, 31]. Currently, male sexual function is comprehensively analyzed and mechanistically understood based on several studies [30, 33], whereas female dysfunction is only based on theoretical considerations and underrepresented in literature. On the one hand, sympathetic nerve injury leads to ejaculatory problems in men [31], such as absent, retrograde, or painful ejaculation, and can induce vaginal dryness, diminished inner genital sensation, and orgasm disorders in female patients [30]. On the other, erectile dysfunction [31] due to impairment of vasodilatory function of the erectile tissue and reduced labial swelling [30] are caused by parasympathetic neural damage.

In our series, erectile function (p = 0.002), satisfaction with sexual intercourse (p = 0.034), and IIEF final score (p = 0.003) deteriorated significantly after TaTME in male patients, while RoTME led to stable pre- and postoperative IIEF scores. For female patients, sexual desire decreased significantly after TaTME (p < 0.001), but no significant changes were detectable postoperatively in the RoTME group. After adjustment for confounders, male sexual function was comparable between techniques. For female patients, only pain during sexual intercourse was significantly increased for RoTME patients.

## Limitations

RoTME and TaTME were performed in different hospitals by different surgeons. To establish an adequate level of quality, only patients treated after the surgeons had completed the learning curve of at least 40 cases in the respective technique were included. Therefore, only one approach was eligible per hospital and surgeon. Nevertheless, this fact might bias the observations. Moreover, age, proportion of neoadjuvant therapy and surgical procedures differed significantly between groups. To balance these effects, score differences were analyzed after adjustment for these confounders. Since patient age is an unspecific risk factor [42], preexisting functional status was recognized by baseline score values. However, this is a limitation of our study.

## Conclusion

Our study represents the first direct functional comparison of RoTME and TaTME and incorporates the largest series of TaTME cases on functional outcome. Overall, RoTME and TaTME resulted in only mild functional impairment after rectal resection. RoTME led to better anorectal results, potentially due to sphincter dilatation or damage of the internal sphincter during TaTME. Meanwhile, there was a noticeably lower rate of severe LARS after both TaTME and RoTME than previously published, despite frequent risk factors such as chemoradiation and low anastomosis. Urinary function benefitted from TaTME in male patients and showed advantages for female patients. Optimized visualization of the hypogastric nerves might support favorable urinary function after TaTME. With surgery having the least impact on sexual function, the results appear comparable for RoTME and TaTME. Our study provides the first evidence of advantages of advanced minimally invasive TME techniques on long-term functional outcomes for rectal cancer patients. Further high-quality analyses are needed to corroborate this observation.

## Supplementary Information

Below is the link to the electronic supplementary material.

Supplementary file1 (PDF 134 KB)

Supplementary file2 (DOCX 35 KB)

## Data Availability

Data are available from the corresponding author upon request.
