# Supplementary material for: Robotic versus transanal total mesorectal excision in sexual, anorectal, and urinary function: a multicenter, prospective, observational study
Source: Int J Colorectal Dis. 2021 Sep 18;36(12):2749–61. doi: 10.1007/s00384-021-04030-5 (PMC8589758; doi:10.1007/s00384-021-04030-5)
Supplement: Supplementary file 1 — Supplementary file1 (PDF 134 KB) [file 384_2021_4030_MOESM1_ESM.pdf]

Online Resource Fig. 1 Additional scores for urinary function measured: ICIQ-MLUTS for male patients and ICIQ-FLUTS for female patients. Favorable results are at the lower end of the scale.

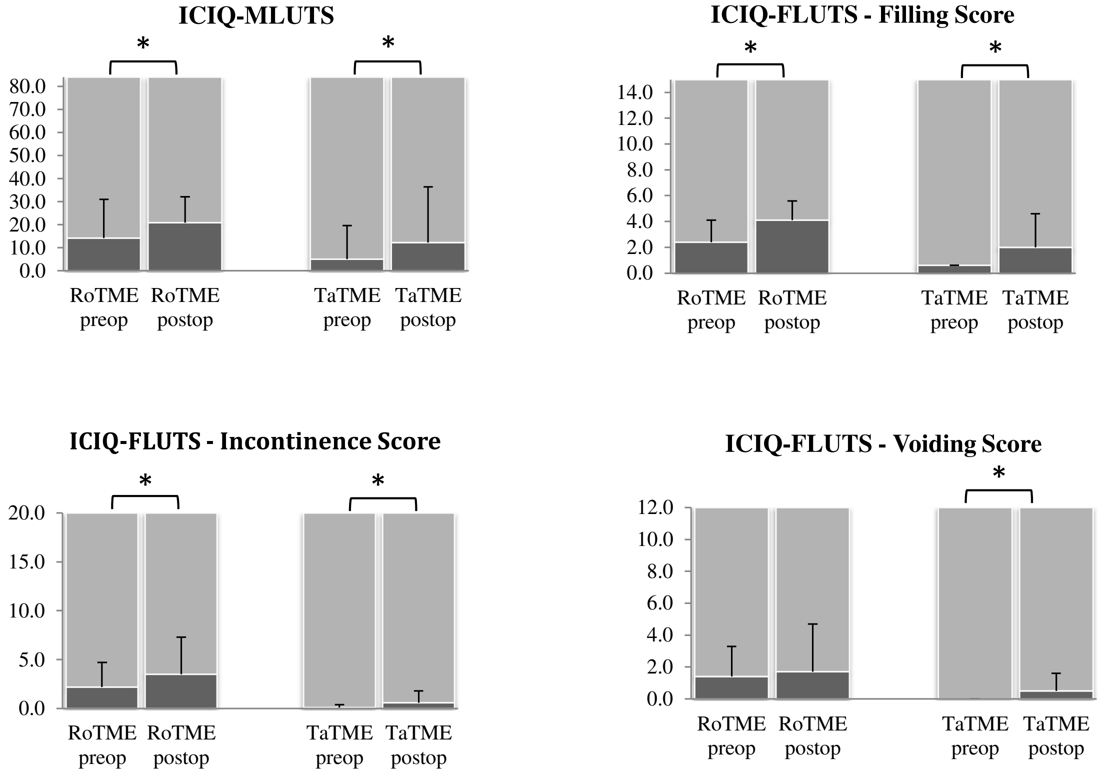

	RoTME			TaTME			Range
	Preop.	Postop.	p-value	Preop.	Postop.	p-value	
Male							
**ICIQ-MLUTS LF**	14.2±16.8	20.9±11.2	**0.032**	5.0±14.6	12.2±24.2	**<0.001**	1-84
Female							
**ICIQ-FLUTS Filling Score**	2.4±1.7	4.1±1.5	**0.017**	0.6±0.9	2.0±2.6	**0.007**	0-15
**ICIQ-FLUTS Incontinence Score**	2.2±2.5	3.5±3.8	**0.027**	0.1±0.3	0.6±1.2	**0.041**	0-20
**ICIQ-FLUTS Voiding Score**	1.4±1.9	1.7±3.0	0.480	0.0±0.0	0.5±1.1	**0.027**	0-12

Numbers indicated as mean ± standard deviation, p-values in bold indicate statistical significance between surgical techniques and are indicated on the figure as *. ICIQ-MLUTS/ICIQ-FLUTS, International Consultation on Incontinence – Male/Female Lower Urinary Tract Symptoms Score; LF, long form; RoTME – robotic total mesorectal excision; SD – standard deviation; TaTME – transanal total mesorectal excision.

Robotic versus transanal total mesorectal excision in sexual, anorectal and urinary function: a multicenter study

International Journal of Colorectal Disease

Julia-Kristin Grass *, Roberto Persiani, Flavio Tirelli, Chien-Chih Chen, Marco Caricato, Alice Pecorino, Isabelle J Lang, Marius Kemper, Jakob R Izbicki, Nathaniel Melling, Daniel Perez

*Department of General, Visceral and Thoracic Surgery, University Medical Centre of Hamburg-Eppendorf, Hamburg, Germany; j.grass@uke.de

Online Resource Fig. 2 Additional results for male sexual function measured by IIEF. Favorable results are at the upper end of the scale.

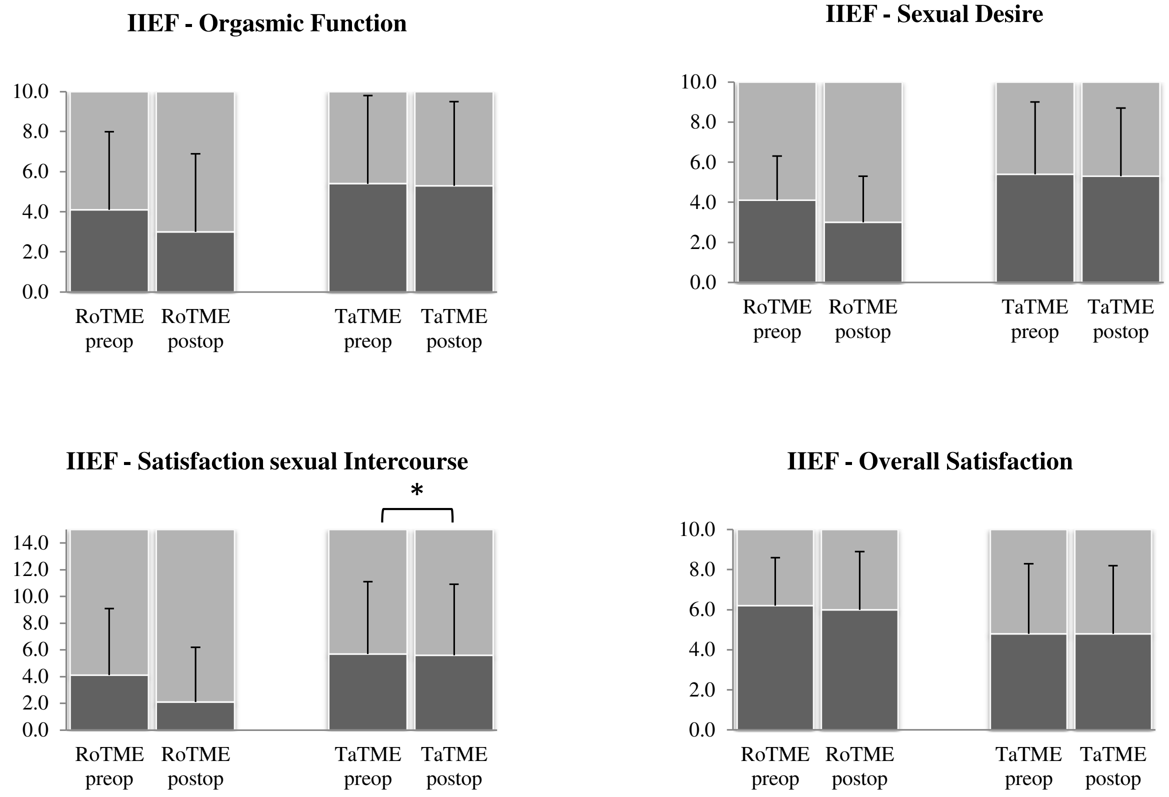

	RoTME			TaTME			Range
	Preop.	Postop.	**p-value**	Preop.	Postop.	**p-value**	
**IIEF - Orgasmic Function**	4.1±3.9	3.0±3.9	0.574	5.4±4.4	5.3±4.2	0.531	0-10
**IIEF - Sexual Desire**	4.8±2.2	3.9 ± 2.3	0.168	4.9±3.6	4.8±3.4	0.279	0-10
**IIEF - Satisfaction Sexual Intercourse**	4.1 ± 5.0	2.1 ± 4.1	0.116	5.7±5.4	5.6±5.3	**0.034**	0-15
**IIEF - Overall Satisfaction**	6.2±2.4	6.0±2.9	0.674	4.8±3.5	4.8±3.4	0.785	0-10

Numbers indicated as mean ± standard deviation, p-values in bold indicate statistical significance between surgical techniques and are indicated on the figure as *. IIEF, International Index of Erectile Function; RoTME, robotic total mesorectal excision; TaTME, transanal total mesorectal excision.

Robotic versus transanal total mesorectal excision in sexual, anorectal and urinary function: a multicenter study

International Journal of Colorectal Disease

Julia-Kristin Grass *, Roberto Persiani, Flavio Tirelli, Chien-Chih Chen, Marco Caricato, Alice Pecorino, Isabelle J Lang, Marius Kemper, Jakob R Izbicki, Nathaniel Melling, Daniel Perez

*Department of General, Visceral and Thoracic Surgery, University Medical Centre of Hamburg-Eppendorf, Hamburg, Germany; j.grass@uke.de

Online Resource Fig. 3 Additional subscores for female sexual function measured by FSFI. Favorable results are at the upper end of the scale.

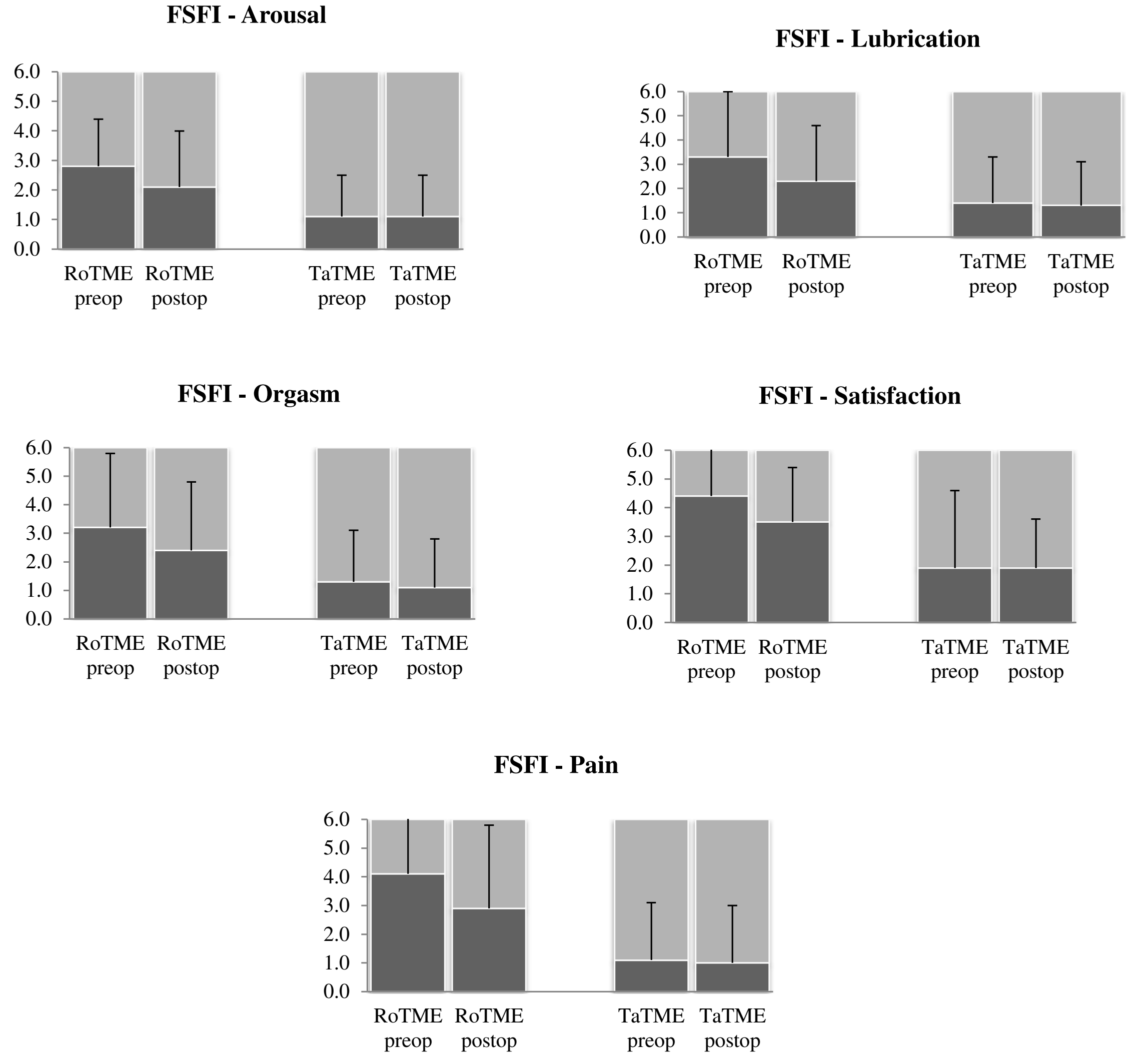

	RoTME			TaTME			Range
	Preop.	Postop.	p-value	Preop.	Postop.	p-value	
FSFI - Arousal	2.8±1.6	2.1±1.9	0.540	1.1±1.4	1.1±1.4	0.906	0-6
FSFI - Lubrication	3.3±2.7	2.3±2.3	0.284	1.4±1.9	1.3±1.8	0.180	0-6
FSFI - Orgasm	3.2±2.6	2.4±2.4	0.440	1.3±1.8	1.1±1.7	0.180	0-6
FSFI - Satisfaction	4.4±1.7	3.5±1.9	0.342	1.9±1.7	1.9±1.7	0.317	0.8-6
FSFI - Pain	4.1±2.7	2.9±2.9	0.219	1.1±2.0	1.0±2.0	0.655	0-6

Numbers indicated as mean ± standard deviation, p-values in bold indicate statistical significance between surgical techniques and are indicated on the figure as *.
FSFI, Female Sexual Function Index; RoTME, robotic total mesorectal excision; TaTME, transanal total mesorectal excision.

Robotic versus transanal total mesorectal excision in sexual, anorectal and urinary function: a multicenter study

International Journal of Colorectal Disease

Julia-Kristin Grass *, Roberto Persiani, Flavio Tirelli, Chien-Chih Chen, Marco Caricato, Alice Pecorino, Isabelle J Lang, Marius Kemper, Jakob R Izbicki, Nathaniel Melling, Daniel Perez

*Department of General, Visceral and Thoracic Surgery, University Medical Centre of Hamburg-Eppendorf, Hamburg, Germany; j.grass@uke.de
